# Log-periodic quantum magneto-oscillations and discrete-scale invariance in topological material HfTe_5_

**DOI:** 10.1093/nsr/nwz110

**Published:** 2019-08-06

**Authors:** Huichao Wang, Yanzhao Liu, Yongjie Liu, Chuanying Xi, Junfeng Wang, Jun Liu, Yong Wang, Liang Li, Shu Ping Lau, Mingliang Tian, Jiaqiang Yan, David Mandrus, Ji-Yan Dai, Haiwen Liu, Xincheng Xie, Jian Wang

**Affiliations:** 1 International Center for Quantum Materials, School of Physics, Peking University, Beijing 100871, China; 2 Department of Applied Physics, The Hong Kong Polytechnic University, Hong Kong, China; 3 Wuhan National High Magnetic Field Center, Huazhong University of Science and Technology, Wuhan 430074, China; 4 High Magnetic Field Laboratory, Chinese Academy of Sciences, Hefei 230031, China; 5 Center of Electron Microscopy, State Key Laboratory of Silicon Materials, School of Materials Science and Engineering, Zhejiang University, Hangzhou 310027, China; 6 Materials Science and Technology Division, Oak Ridge National Laboratory, Oak Ridge, TN 37831, USA; 7 Department of Materials Science and Engineering, University of Tennessee, Knoxville, TN 37996, USA; 8 Center for Advanced Quantum Studies, Department of Physics, Beijing Normal University, Beijing 100875, China; 9 Collaborative Innovation Center of Quantum Matter, Beijing 100871, China; 10 CAS Center for Excellence in Topological Quantum Computation, University of Chinese Academy of Sciences, Beijing 100190, China; 11 Beijing Academy of Quantum Information Sciences, Beijing 100193, China

**Keywords:** log-periodic oscillations, discrete-scale invariance, topological materials, Dirac materials, magnetoresistance, Hall resistance

## Abstract

Discrete-scale invariance (DSI) is a phenomenon featuring intriguing log-periodicity that can be rarely observed in quantum systems. Here, we report the log-periodic quantum oscillations in the longitudinal magnetoresistivity (*ρ_xx_*) and the Hall traces (*ρ_yx_*) of HfTe_5_ crystals, which reveal the DSI in the transport-coefficients matrix. The oscillations in *ρ_xx_* and *ρ_yx_* show the consistent log*B*-periodicity with a phase shift. The finding of the log*B* oscillations in the Hall resistance supports the physical mechanism as a general quantum effect originating from the resonant scattering. Combined with theoretical simulations, we further clarify the origin of the log-periodic oscillations and the DSI in the topological materials. This work evidences the universality of the DSI in the Dirac materials and provides indispensable information for a full understanding of this novel phenomenon.

## INTRODUCTION

Discrete-scale invariance (DSI) is a partial breaking of continuous-scale invariance where observables of the system obey the scale invariance only for a geometrical set of choices written in the form of λ*^n^*, with λ being the scaling ratio [1]. With the violation of the classical continuous-scale symmetry, the DSI represents a scale anomaly and the characteristic signature of DSI, the intriguing log-periodicity, exists in rupture, growth processes, turbulence, finance and so on. The appearance of log-periodic structures indicates the characteristic length scales in a system, which is extremely interesting when it is fundamentally related to the underlying physical mechanism [1].

The scale anomaly DSI is of high general interest while it can be rarely observed in quantum systems experimentally [2]. For a long time, the DSI has only been confirmed in cold atom systems and generated tremendous interest [3–10]. Nowadays, the DSI behavior in Dirac materials has also attracted attention in several subfields of physics [11–16]. Especially, the magneto-transport measurements on topological material ZrTe_5_ reveal a new type of magnetoresistance (MR) oscillations with peculiar log-periodicity and thus manifest the appearance of DSI in a solid-state system [14]. Such a peculiar DSI feature is considered to be universal in Dirac materials with Coulomb attraction [14], which may be closely related to the quasi-bound states formed by massless Dirac fermions and the long-pursued atomic-collapse phenomenon [14,15,17]. Thus, it is desirable to explore the log-periodic quantum oscillations and the DSI in other physical observables, such as the Hall trace, and the comparison of the DSI features in different transport coefficients may provide insights into the underlying mechanisms. As a sister compound of ZrTe_5_, the topological material HfTe_5_ provides a promising platform [18–32].

**Figure 1:**
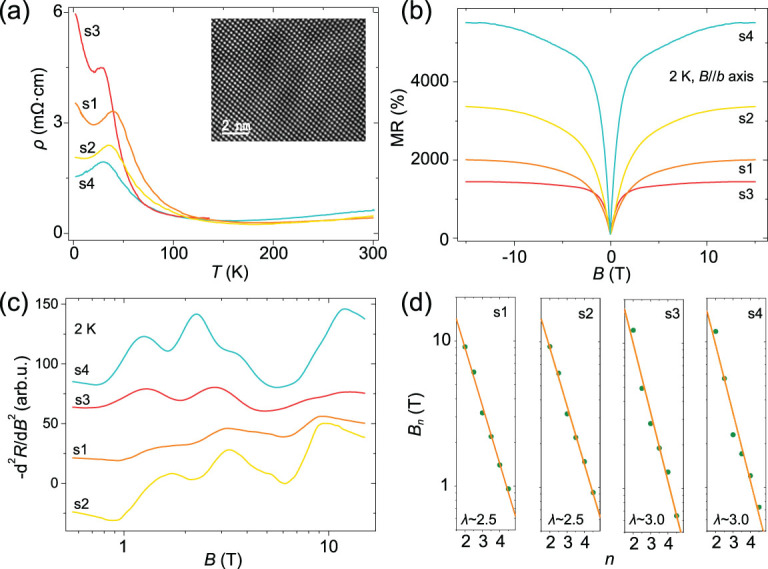
Resistivity-temperature characteristic and MR behavior of HfTe_5_ single crystals. (a) Temperature dependence of the resistivity. Inset: the atomically high-resolution transmission-electron-microscopy image of HfTe_5_ manifesting a high-quality nature. (b) MR at 2 K for HfTe_5_ crystals in a perpendicular magnetic field. (c) The second derivative results of the MR behavior at 2 K shown in (b). Data curves are shifted for clarity. (d) Linear dependence of log*B_n_* on the index *n* shows log-periodicity of the MR oscillations. *B_n_* is the characteristic magnetic field for a peak or dip in the oscillations.

In this work, we reveal the universality of the peculiar log-periodic quantum oscillations and DSI phenomenon in Dirac materials by the magneto- transport results of HfTe_5_ crystals. The oscillations with log*B*-periodicity are demonstrated in the MR behavior, almost independently of the minor differences of the sample quality. More importantly, the log*B*-periodicity is also discovered in the Hall traces of the HfTe_5_ crystals. In addition, we observe a phase shift in the oscillations of *ρ_xx_* and *ρ_yx_* with consistent period, justifying the log*B*-periodic oscillations originating from the resonant scattering around the Fermi energy. Moreover, we elaborate on the relation between the DSI and the log-periodic oscillations in both the longitudinal MR and the Hall resistance, and explain the origin of the log*B*-periodic oscillations and the phase shift between *ρ_xx_* and *ρ_yx_*. This work provides new insights towards further understanding of the log-periodic quantum oscillations and the DSI in solid-state systems.

## RESULTS

### Magnetoresistance behavior

Single crystals HfTe_5_ in our work were grown via a self-Te-flux method as in the previous report [23]. The atomically high-resolution transmission-electron-microscopy image of one typical sample is shown in the inset of Fig. 1a, which manifests a high-quality nature. The resistivity-temperature (*ρT*) characteristic of HfTe_5_ crystals down to 2 K is shown in Fig. 1a. With decreasing temperatures, the samples show first the metallic behavior above approximately 200 K and then a semiconducting-like upturn. As the temperature is further decreased, sample-dependent resistivity peaks are observed at temperatures *T*_p_ varying from 20 to 40 K. At even lower temperatures, the semiconducting-like upturn recovers in most samples. It is noted here that the resistivity peak in the crystals cannot be attributed to Lifshitz transition, since the Hall remains positive up to room temperature with no sign change [23].

Figure 1b shows the MR behavior at 2 K of different samples from the same batch when the magnetic field is perpendicular to the layer orientation (B//*b* axis). The MR follows a sharp cusp at around zero magnetic field and changes much more slowly at high magnetic fields. The MR (*R*(H)/*R*(0)) values show sample dependence with a range of 1500–5500% at 15 T. According to the non-linear Hall data of HfTe_5_ crystals, we would attribute the various *ρT* behavior and MR effect to the competition of a semi-metallic Dirac band and a semiconducting band in the material [33]. For Sample 3 (s3), the Fermi level is very close to the Dirac point and thus the Fermi surface of the Dirac pocket is tiny. The dominated semiconducting band induces the upturn resistance at low temperatures and the MR effect is small. On the contrary, the Dirac band dominates in s4, which gives rise to the metallic *ρT* behavior at low temperatures down to 2 K and the larger MR effect in s4. For the other two samples (s1 and s2), they exhibit the intermediate properties as shown in Fig. 1a and b. Since the transition-metal pentatelluride system is extremely sensitive to the cell volume, the slight diversity of these transport properties could be attributed to the minor quality differences in the samples. A similar two-band model was proposed to interpret the mysterious peak in ZrTe_5_ considering either Te deficiency or iodine contamination [34,35]. The model might give a unified explanation for the various observations of the Dirac or semiconducting property in ZrTe_5_.

**Figure 2:**
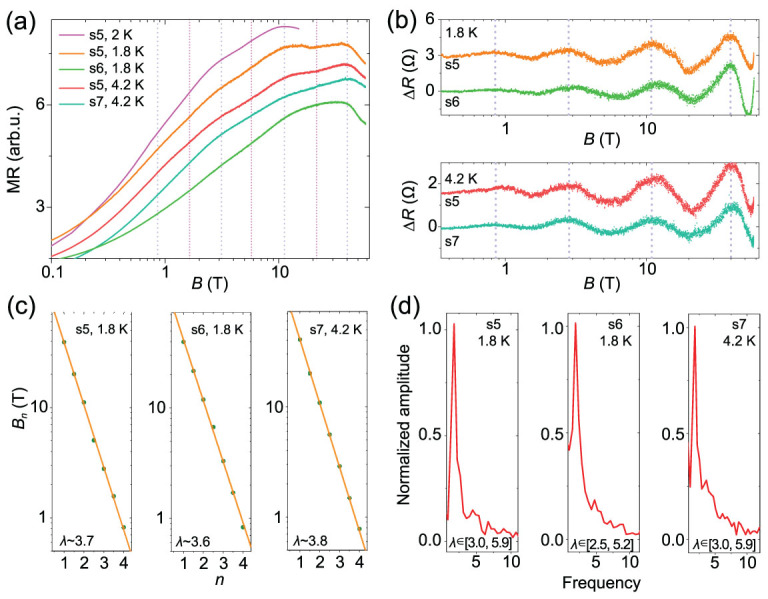
Log-periodic MR oscillations in HfTe_5_. (a) MR of HfTe_5_ vs. log*B*. The MR oscillations measured in PPMS (pink) is consistent with the results (orange and red) in the pulsed high magnetic field. MR oscillations are reproduced in different samples (s5, s6, s7). Dashed lines serve as guides to the eye. (b) Extracted MR oscillations from the raw data in (a) after subtracting a background. Data curves in (a) and (b) are shifted for clarity. (c) Log*B*-periodicity of the MR oscillations in HfTe_5_. (d) FFT results of the MR oscillations in (b). Combining the results of different samples, the scale factor λ shows a range of about [2.5, 5.9] which is determined by the FWHM of the FFT frequency peak.

By performing the second derivative for the MR results in Fig. 1b, oscillations can be distinguished from the large MR background. The characteristic magnetic fields *B_n_* of oscillating peaks (marked with index *n*) and dips (*n*-0.5) in the oscillations are approximately consistent for different HfTe_5_ samples (Fig. 1c and Supplementary Fig. S1). By plotting log*B_n_* vs. *n* in Fig. 1d, the index dependence for different samples can all be reproduced by a linear fitting, which reveals that peaks and dips appear periodically as a function of log*B* in HfTe_5_. We identify that these specific magnetic fields satisfy the law of *B_n_* = λ*·B*_*n* + 1_, where λ is a characteristic scale factor for the material. From the index plot in Fig. 1d, the dominant scale factor λ is shown to be 2.5 or 3.0.

The magneto-transport measurements at high magnetic fields up to 58 T further confirm the log*B*-periodic MR oscillations and DSI in HfTe_5_ (Fig. 2 and Supplementary Fig. S2). For clarity, data curves in Fig. 2a and b are shifted. The pink curve for s5 in Fig. 2a is measured at a static magnetic field. The MR oscillations observed at lower magnetic fields can be well reproduced by the pulsed magnetic field measurements on s5 and more oscillations are observed at higher magnetic fields (orange and red), as guided by the dashed lines. Besides, the oscillations are also observed in other samples s6 and s7, where the resistance peaks and dips in s5 can be replicated. The oscillations can be extracted by subtracting a smooth background from the raw data in Fig. 2a and the results are shown in Fig. 2b. The consistent log*B*-periodicity can also be obtained by the second derivative of the raw MR data (see Supplementary Fig. S3). The index plots for the oscillations are shown in Fig. 2c, which confirms the log*B*-periodicity of the MR oscillations with more experimental points at ultrahigh magnetic fields (green dots). By performing the Fast Fourier Transform (FFT) of the log-periodic oscillations in Fig. 2b, a sharp FFT frequency peak is observed in various samples (Fig. 2d), which is consistent with the linear fitting results shown in Fig. 2c. It is worth noting that the factor λ has a broadening width in experiments. Based on an error bar determined by the full width at half maximum (FWHM) of the FFT frequency peak and combining with the results of different samples, we obtain a factor range of about [2.5, 5.9] in the HfTe_5_ crystals.

### Temperature dependence

**Figure 3:**
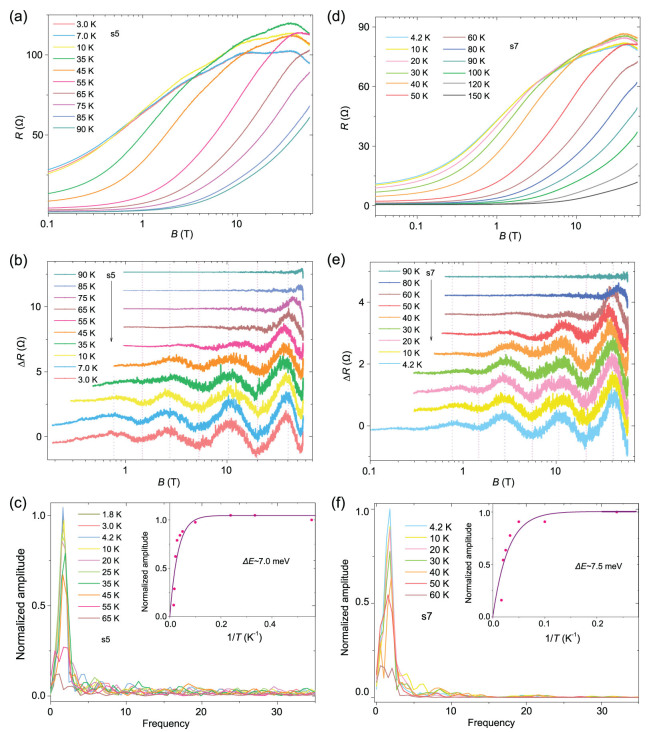
Temperature dependence of the log*B*-periodic oscillations. (a) MR of HfTe_5_ (s5) at selected temperatures. (b) Log*B*-periodic oscillations in s5 at selected temperatures. (c) FFT results for the MR oscillations in (b). Inset: Theoretical fit on the normalized FFT amplitude at varying temperatures based on the theoretical formula *A* = *A*_0_(1-exp(−∆*E*/*k*_B_*T*)). The fitted value is consistent with the experimental observations. (d)–(f) are results for another sample (s7). The fitting parameter is also consistent with experiments. The fitting results indicate that the disappearance temperature of the oscillations in the HfTe_5_ crystal is about 80–90 K. Data curves in (b) and (e) are shifted for clarity.

The temperature dependence of the log*B*-periodic oscillations in HfTe_5_ is shown in Fig. 3. Figure 3a–c are results for s5 and Fig. 3d–f are results for another sample (s7). By subtracting background from the raw MR data in Fig. 3a and d, we obtain the oscillating resistance shown in Fig. 3b and e, respectively, and then perform FFT of the oscillations. The FFT amplitudes in Fig. 3c and f are normalized divided by the peak amplitude at the base temperature. Based on the theoretical formula of *A* = *A*_0_(1-exp(−∆*E*/*k*_B_*T*)), the fitting of the FFT amplitude at varied temperatures gives a characteristic binding energy ∆*E* of 7.0 meV for s5 and 7.5 meV for s7. Here, ∆*E* refers to the binding energy of the states that induce log-periodic oscillations. It means that, when the energy is larger than this characteristic value, the states dissolve. The binding energies correspond to the disappearance temperatures of the oscillations equaling to 81 and 87 K for s5 and s7, respectively. The characteristic temperatures for both samples are consistent with our experimental observations.

**Figure 4:**
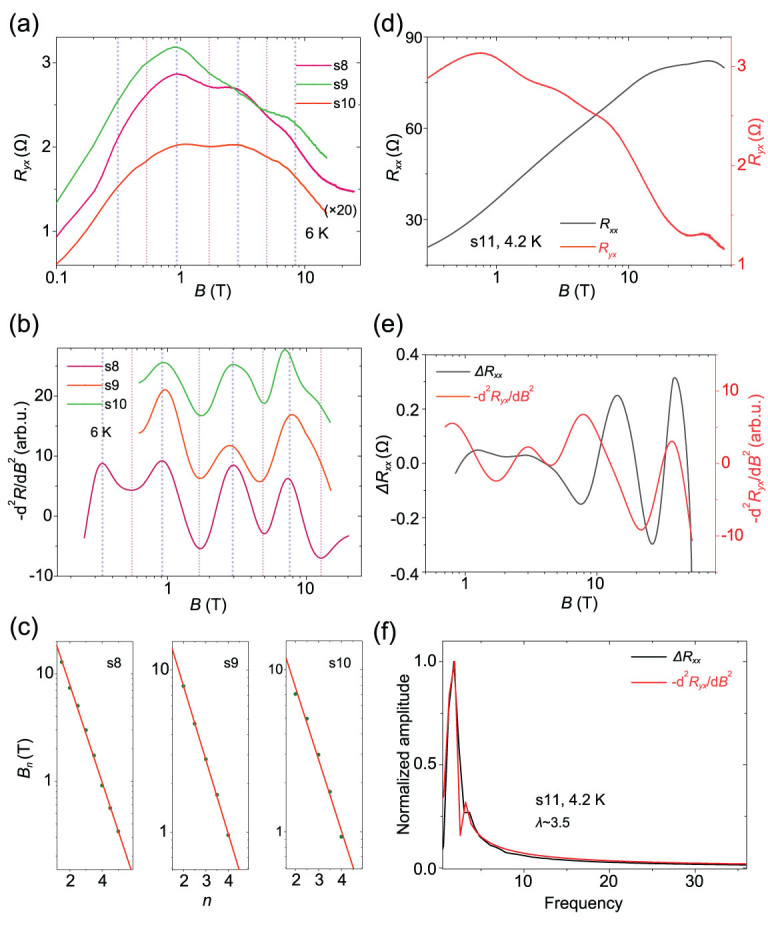
Signals of log*B*-periodic oscillations in the Hall traces of HfTe_5_. (a) Hall traces of HfTe_5_ crystals at 6 K versus the magnetic field. (b) The second derivative results of the curves in (a). Data curves are shifted for clarity. (c) The log-periodicity of the oscillating Hall resistance. (d) Comparison of *R_xx_* and *R_yx_* in the same sample (s11). (e) Comparison of the oscillations in *R_xx_* and *R_yx_* in the same sample s11. (f) FFT results for the oscillations in *R_xx_* and *R_yx_*.

### Oscillations in Hall resistance

We finally investigated the influence of the DSI on the Hall traces of HfTe_5_. In Fig. 4a, the Hall data of HfTe_5_ clearly shows a non-linear dependence on the magnetic field, which is consistent with the two-band model. Distinct and consistent oscillations are observed in the Hall resistance of different samples. The second derivate results are shown in Fig. 4b. Similar to the property of oscillations on the MR, the characteristic log*B*-periodicity of the Hall resistance oscillations is confirmed by the linear index dependence (Fig. 4c). Here, the finding of the log*B* oscillations in the Hall resistance is quite meaningful, since it can identify the log*B* phenomenon in the total transport coefficients as a general quantum effect. We further compared the characteristic magnetic fields *B_n_* where the peaks and valleys appear in the MR and the Hall resistance. Figure 4d and e shows the longitudinal MR (*R_xx_*) and Hall results (*R_yx_*) on the same sample (s11). The extracted oscillations from the *R_xx_* and *R_yx_* are shown in Fig. 4e. The overlapped frequency peaks in Fig. 4f indicate the consistent log*B*-periodicity in the behavior of MR and Hall. Moreover, it is found that, in the oscillations, the phase of *R_yx_* is slightly ahead of *R_xx_*. The phase difference between *R_yx_* and *R_xx_* is reminiscent of that in the 2D quantum Hall effect [36]. Thus, the log-periodic quantum magneto-oscillations in both the longitudinal MR and the Hall traces indicate the underlying DSI property of the topological material HfTe_5_ and its evolution under the magnetic field.

## DISCUSSION

As discussed previously [14], the log-periodic quantum magneto-oscillations cannot be attributed to the conventional quantum oscillations, such as the Shubnikov–de Haas oscillations even with the consideration of the Zeeman effect. In addition, the peculiar phenomenon shows different features compared with the field-induced Fermi surface deformation or reconstruction scenario, such as the density-wave transition. It is suggested that log-periodic oscillations are closely related to the quasi-bound states of Weyl particles from Coulomb attraction (see Supplementary Fig. S4) [14] and the resonant scattering between the mobile carriers and the quasi-bound states around the Fermi level determines the DSI features in both the longitudinal MR and the Hall traces.

The oscillation term in *ρ_xx_* and *ρ_yx_* can be obtained by the T-matrix approximation and the theoretical quantum magneto-oscillations curves in *ρ_xx_* and *ρ_yx_* are shown in Supplementary Fig. S5. We can see that both *ρ_xx_* and *ρ_yx_* satisfy the log-periodic property. Furthermore, the peaks of *Δρ_xx_* correspond to the nodes of *Δρ_yx_*, indicating a π/2 phase shift in the oscillations of *ρ_xx_* and *ρ_yx_*. The theoretical formulas signify that the π/2 phase shift originates from the resonant scattering between the mobile carriers and the quasi-bound states, sharing the same origin of the log-periodic oscillations. The experimental observations of *R_xx_* and *R_yx_* reveal that the phase of *R_yx_* is slightly ahead of *R_xx_*, consistently with the theoretical simulations. Here, we point out that the oscillations can be qualitatively viewed as a density-of-states effect, which refers to increased density of states when the quasi-bound states evolve into the Fermi energy. However, the quantitative understanding of the DSI in *ρ*_xx_ and *ρ*_yx_ and their phase shift needs the introduction of scattering. Since the quasi-bound states cannot transfer charge, these quasi-bound states contribute to the transport property by scattering with mobile carriers when the quasi-bound states locate at the Fermi energy. Previously, the 2D quantum Hall effect [36] also shows the π/2 phase shift between *ρ_xx_* and *ρ_yx_* when changing the magnetic field, which can also be explained by the scattering mechanism.

The correction to *ρ_xx_* is in the order *n_C_*/*n_S_*, while that to *ρ_yx_* is in the order *n_C_*/*N*; here, *n_C_*, *n_S_* and *N* denote the density of the quasi-bound states, the density of short-range impurity and the total carrier density, respectively. Thus, the log-periodic magneto-oscillations only occupy a small percentage of the total resistance due to the small ratio of *n_C_*/*n_S_* and *n_C_*/*N*. In HfTe_5_, the oscillating resistance is about 0.4–0.9% of the longitudinal MR for the *n* = 2 peak as shown in Fig. 3 and the oscillations in the Hall resistance are more apparent (about 2%) as shown in Fig. 4. When comparing the results of HfTe_5_ with those of ZrTe_5_ [14], we find two differences. First, the oscillating amplitude of the log-periodic quantum oscillations in *ρ_xx_* of HfTe_5_ is relatively smaller. We attribute the phenomenon to a large *n_S_* and relatively small *n_C_*/*n_S_*; thus, the correction to *ρ_xx_* is weak. Meanwhile, due to the relatively large impurity scattering from these short-range impurities, the broadening effect smears the log-periodic oscillations at small magnetic fields, ultimately leading to fewer observable oscillating cycles. Second, the log-periodicity in *ρ_yx_* of HfTe_5_ is relatively more remarkable. This feature may be due to the relatively large value for the density of charge impurity and thus relatively large value for the density of quasi-bound states *n_C_* and the ratio *n_C_*/*N*, which gives rise to considerable correction for Hall trace *ρ_yx_* in HfTe_5_.

Besides the consideration shown above, in real systems, other issues can influence the DSI property. First, both a small band gap *Δ* and finite screening length λ*_S_* impose constraint on the DSI feature by introducing an effective low-energy cut-off. But the quasi-bound states with binding energy larger than the band gap *Δ* and radius smaller than λ*_S_* cannot be influenced. Direct estimation gives the largest value of radius *R_C_* and corresponding magnetic length *l_B,C_* satisfying ${R}_c\approx \sqrt{2\cdot{s}_0}\cdot{l}_{B,C}\approx \sqrt{2\cdot{s}_0}\cdot \min (\hslash{v}_F/\Delta , {\lambda}_s).$ Here ${s}_0=\sqrt{{(Z\cdot \alpha )}^2-1}$, with *Z* denoting the number of the central charge and $\alpha$ denoting the fine structure constant. If one sets *Δ* = 4 meV and neglects the screening effect, *R_C_* ≈ 83 nm and the corresponding magnetic field *B_C_* ≈ 0.8 T. Second, in the system with ultralow carrier density, both the charge impurity and the carrier from the electron band can generate the Coulomb attraction [16]. Here, λ is determined by $Z\cdot \alpha$, with $\lambda ={e}^{2\pi /{s}_0}$ [14]. The effective fine structure constant $\alpha =\frac{e^2}{4\pi{\varepsilon}_0\hslash{v}_F}$ depends on the Fermi velocity *v_F_* in Dirac materials and is expected to be universal in one system irrelevant to mobility and carrier density. Moreover, the Coulomb attraction $V(\overrightarrow{R})=\frac{-Z{e}^2}{4\pi{\varepsilon}_0R}$ is determined by the central charge *Ze*, including the charge impurity or the carriers from the electron band. Indeed, λ shows a small difference in different samples (around 3.6, 3.7 and 3.8 for the three samples) and is almost independent of the minor differences in the sample quality, which indicates that the effective charge *Ze* is very close for different samples, and opposite type of carriers or similar charge impurities acting as the charge center. Third, the magnetic field significantly influences a certain *n*-th quasi-bound state when the magnetic length *l_B_* satisfies the relation ${R}_n\approx \sqrt{2\cdot{s}_0}\ {l}_B$ [14]. In HfTe_5_, the oscillation peaks locate in the range [0.8 T, 40 T] and *s_0_* ≈ 4.8; the corresponding radii of the quasi-bound states locate in the range of 90 ~ 10 nm. Finally, a non-oscillating background is subtracted for magneto-oscillations in MR, which is commonly utilized in analysis of quantum oscillations [37,38] and does not influence the DSI feature in our investigations.

## CONCLUSIONS

In summary, we report the intriguing log-periodic quantum magneto-oscillations in both the Hall and the MR results in the topological material HfTe_5_, which indicate the underlying DSI feature in the system. The observation of the log-periodicity in both physical observables *ρ_xx_* and *ρ_yx_* reveals that the DSI shows an overall effect on the transport properties for a Dirac system with the long-range Coulomb attraction. In particular, the finding of the log*B* oscillations in the Hall resistance suggests the general quantum-effect scenario. The origin of the DSI and its relation to the log-periodic magneto-oscillations are further elucidated theoretically. This work paves the way for further research on the log-periodic oscillations and the DSI in quantum systems.

## METHODS

Single crystals of HfTe_5_ in our work were grown via a self-Te-flux method. The crystals were chemically and structurally analysed by powder X-ray diffraction, scanning electron microscopy with energy-dispersive X-ray spectroscopy and transmission electron microscopy. Electrical transport measurements in this work were conducted in three systems: a 16 T- PPMS (Physical Property Measurement System) from Quantum Design, a pulsed high magnetic field facility (58 T) at Wuhan National High Magnetic Field Center and a static magnetic field up to 25 T in the High Magnetic Field Laboratory in Hefei. Results from different measurement systems and different samples are reproducible and consistent with each other. The standard four/six-electrode-method was used for the MR/Hall measurements with the excitation current flowing along the crystallographic *a*-axis of HfTe_5_.

## Supplementary Material

DSI_in_HfTe5-SM-0728_nwz110
