# Supplementary material for: Log-periodic quantum magneto-oscillations and discrete-scale invariance in topological material HfTe5
Source: Natl Sci Rev. 2019 Aug 6;6(5):914–20. doi: 10.1093/nsr/nwz110 (PMC8291527; doi:10.1093/nsr/nwz110)
Supplement: DSI_in_HfTe5-SM-0728_nwz110 [file dsi_in_hfte5-sm-0728_nwz110.docx]

Supplemental Material for

Log-periodic quantum magneto-oscillations and discrete scale invariance in topological material HfTe_5_

Huichao Wang^1,2^^,†^, Yanzhao Liu^1,†^, Yongjie Liu^3^, Chuanying Xi^4^, Junfeng Wang^3^, Jun Liu^5^, Yong Wang^5^, Liang Li^3^, Shu Ping Lau^2^, Mingliang Tian^4^, Jiaqiang Yan^6^, David Mandrus^6,7^, Ji-Yan Dai^2,*^, Haiwen Liu^8,*^, X. C. Xie^1,9,10,11^ and Jian Wang^1,9,10,^^11,*^

*^1^International Center for Quantum Materials, School of Physics, Peking University, Beijing 100871, China*

*^2^Department of Applied Physics, The Hong Kong Polytechnic University, Kowloon, Hong Kong, China*

*^3^Wuhan National High Magnetic Field Center, Huazhong University of Science and Technology, Wuhan 430074, China*

*^4^High Magnetic Field Laboratory, Chinese Academy of Sciences, Hefei 230031, Anhui, China*

*^5^Center of Electron Microscopy, State Key Laboratory of Silicon Materials, School of Materials Science and Engineering, Zhejiang University, Hangzhou, 310027, China ^6^Materials Science and Technology Division, Oak Ridge National Laboratory, Oak Ridge, Tennessee 37831, USA*

*^7^Department of Materials Science and Engineering, University of Tennessee, Knoxville, Tennessee 37996, USA*

*^8^Center for Advanced Quantum Studies, Department of Physics, Beijing Normal University, Beijing, 100875, China*

*^9^Collaborative Innovation Center of Quantum Matter, Beijing 100871, China*

*^10^CAS Center for Excellence in Topological Quantum Computation, University of Chinese Academy of Sciences, Beijing 100190, China*

*^11^Beijing Academy of Quantum Information Sciences, West Bld. #3, No. 10 Xibeiwang East Rd., Haidian District, Beijing 100193, China*

^†^These authors contribute equally to this manuscript

^*^Emails: jianwangphysics@pku.edu.cn (J.W.); [haiwen.liu@bnu.edu.cn](mailto:haiwen.liu@bnu.edu.cn) (H. L.); [jiyan.dai@polyu.edu.hk](mailto:jiyan.dai@polyu.edu.hk) (J.D.)


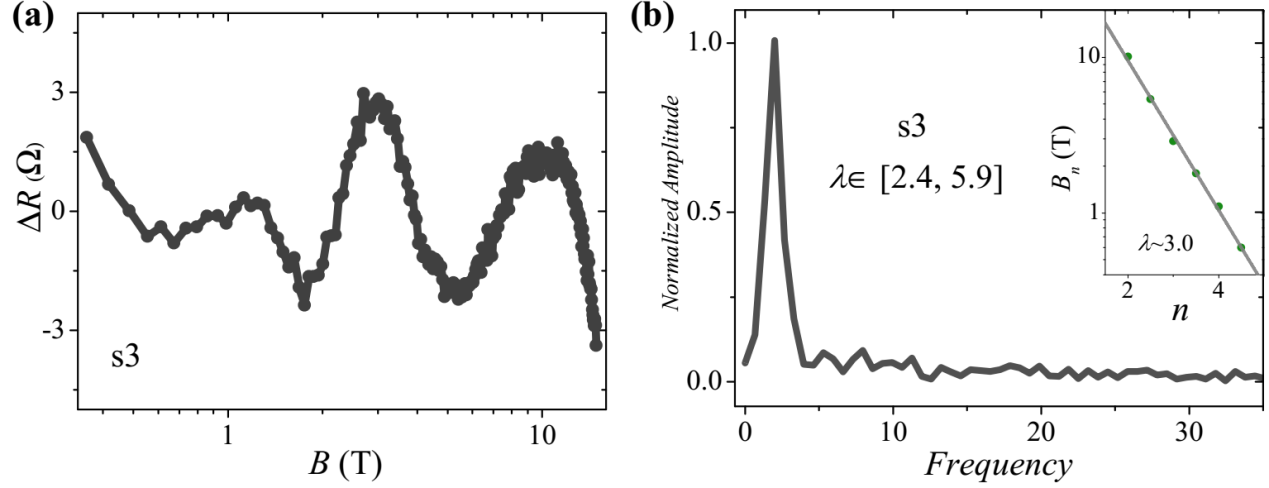


FIG. S1 (a) The magneto-resistance (MR) oscillations in s3 by subtracting a background. (b) The FFT result of (a) confirms the oscillations periodic in log*B*. Inset: the index plot also shows log-periodicity of the oscillations. From the FWHM of the FFT frequency peak, a range of [2.4, 5.9] is obtained for the scale factor *λ*. Thus, the results by subtracting background are consistent with results of the second derivative in Fig.1 in the main text and we demonstrate that the oscillations are intrinsic in HfTe_5_.


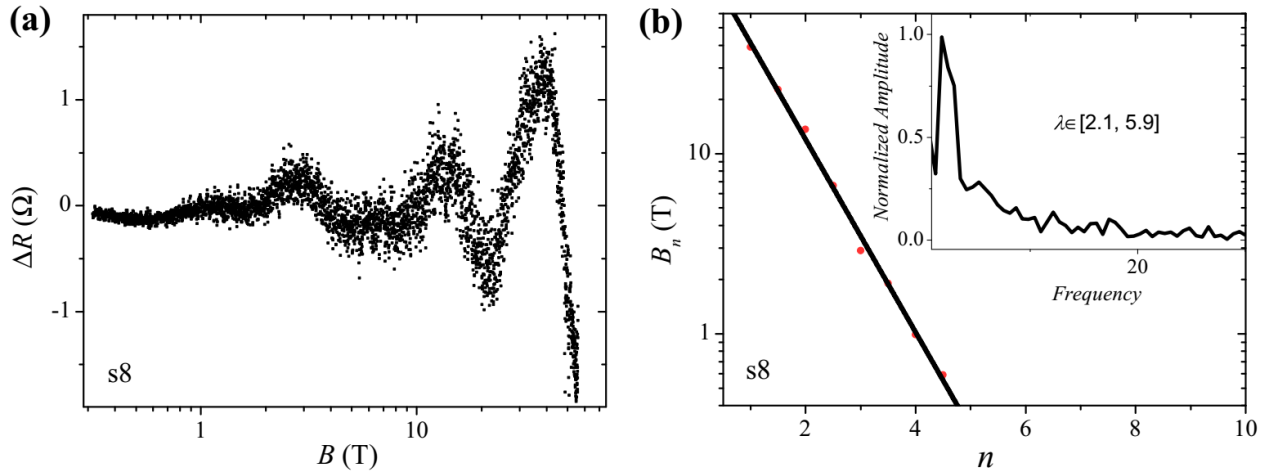


FIG. S2 (a) Extracted MR oscillations in s8 at 4.2 K. (b) The log-periodicity of the MR oscillations. Inset: FFT result of the oscillations in (b). An approximate range for the scale factor is [2.1, 5.9], which is determined by the FWHM of the FFT frequency peak.


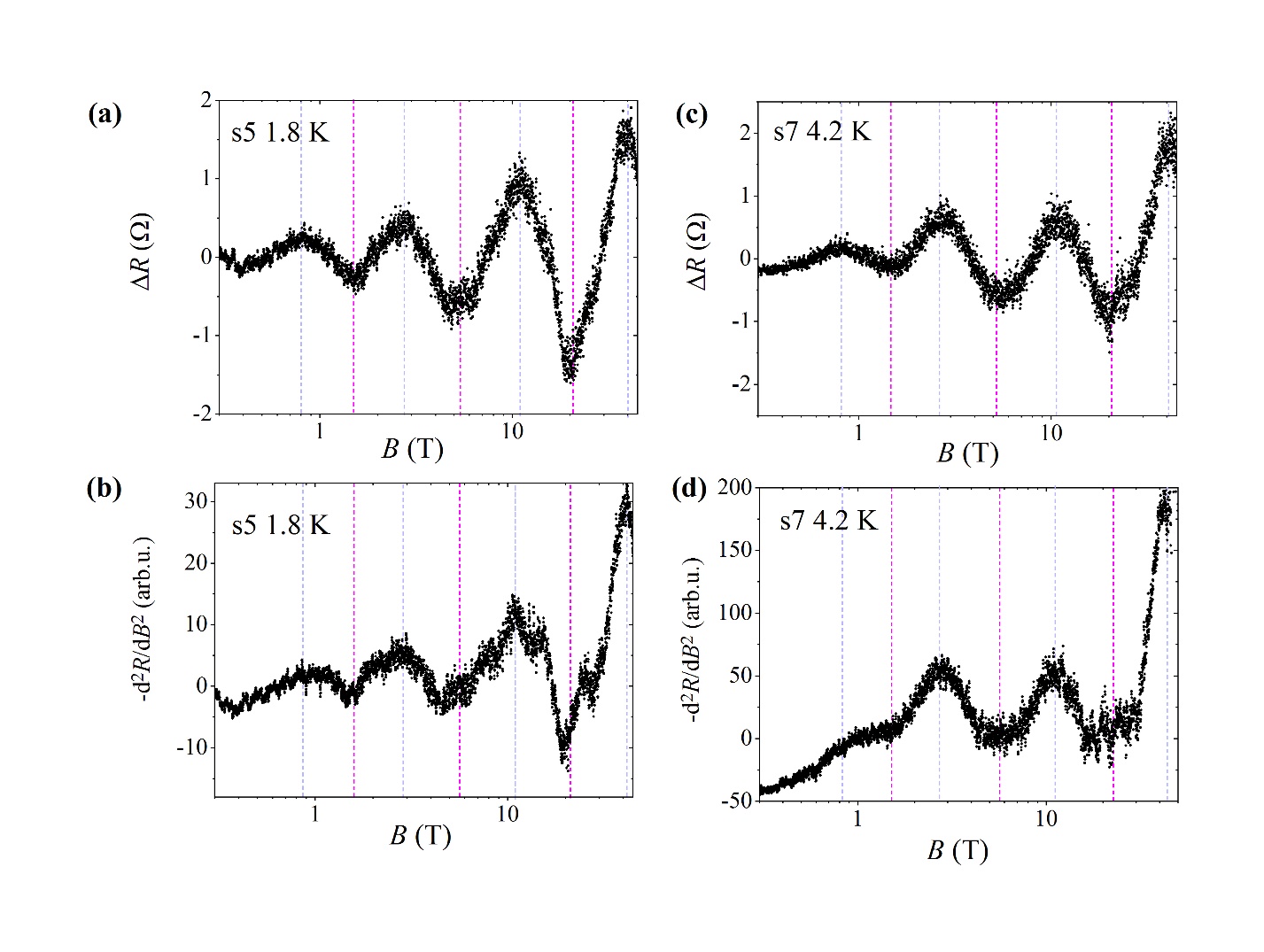


FIG. S3. Extracted log*B*-periodic oscillations. (a) Log*B*-periodic oscillations in s5 at 1.8 K from the raw data after subtracting a smooth background. (b) The second derivative results of the raw MR data of s5 at 1.8 K. (c) Log*B*-periodic oscillations in s7 at 4.2 K from the raw data after subtracting a smooth background. (d) The second derivative results of the raw MR data of s7 at 4.2 K. Dashed lines serve as guides to the eye.


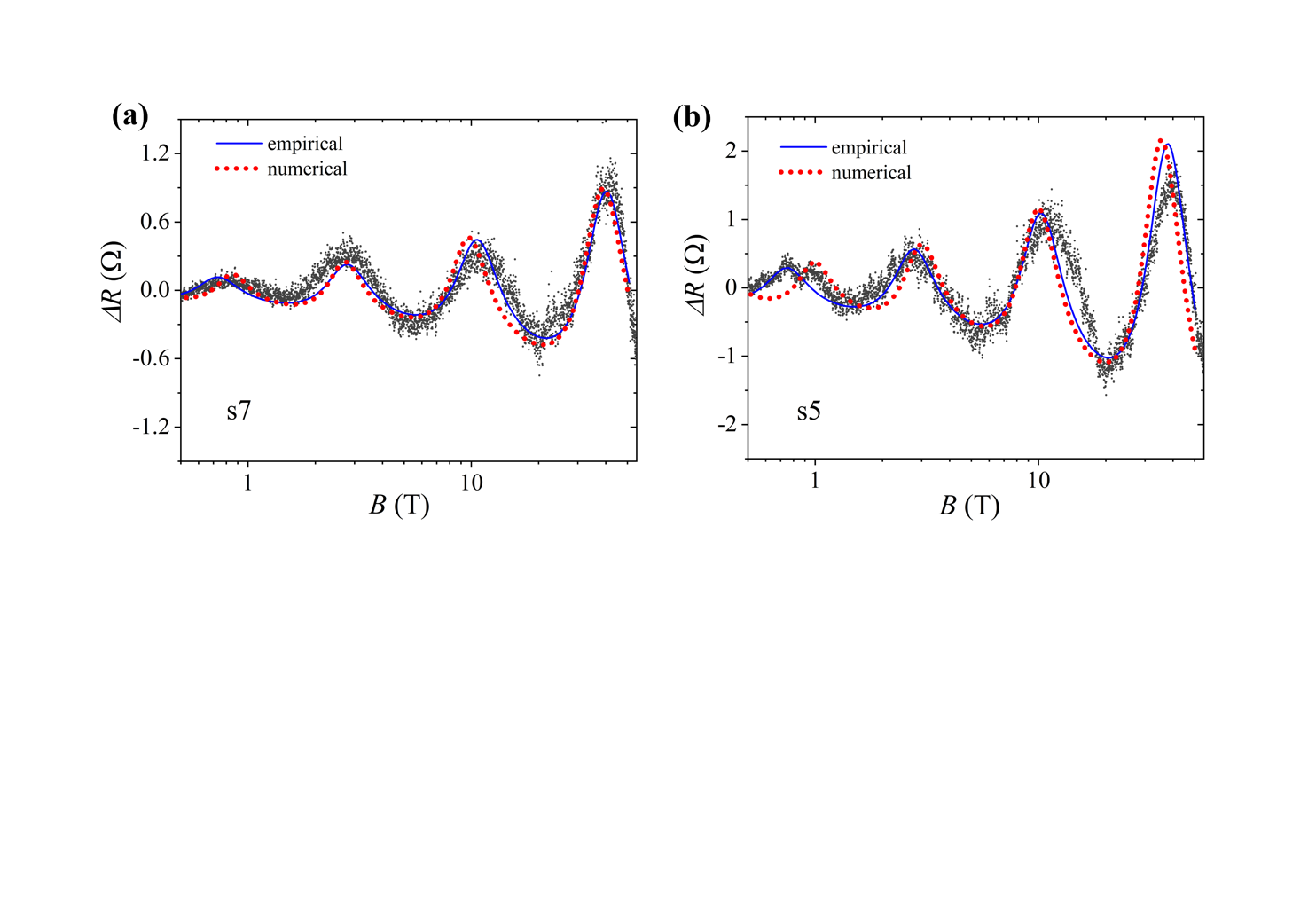


FIG. S4. The fitting of experimental data based on the microscopic numerical calculation and the empirical formula for s7 at 4.2 K (a) and s5 at 3.0 K (b). The fitting parameters are *s*_0_=4.8 (4.7) and *B*_0_=0.20 T (0.19 T) for sample s5 (s7).


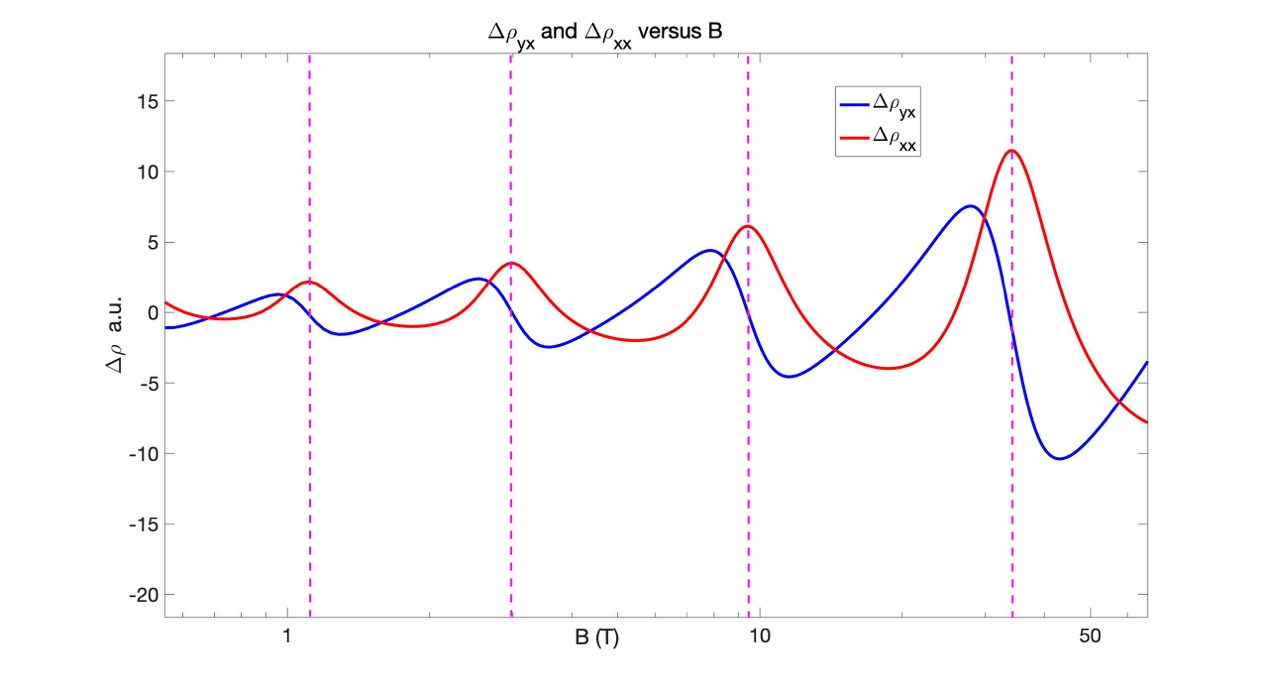


FIG. S5. The theoretical results of log-periodic quantum magneto-oscillations in the Hall resistivity and the longitudinal resistivity. The theoretical curves are obtained by formulas SE1, SE3, SE4 and SE5, and the fitting parameters are *s*_0_=4.6 and *B*_0_=0.20 T. Here, the non-oscillating background is subtracted for both *_yx_* and *_xx_*. The peaks of *_xx_* correspond to the nodes of *_yx_*, which is similar to the feature in two-dimensional quantum Hall effect. The theoretical analysis demonstrates that the resonant scattering between the mobile carriers and the quasi-bound states give rise to the log-periodic oscillations in both *_yx_* and *_xx_* with a π/2 phase shift.

SAMPLE INFORMATION

The samples s1-s4 were only measured in PPMS for magneto-resistance (MR) behavior. The MR of s5 was measured in both the PPMS and the pulsed high magnetic field up to 58 T. The MR of s6 and s7 were only measured in the pulsed high magnetic field. The sample s8 was measured in the pulsed magnetic field up to 58 T to explore the MR oscillations while the Hall trace of s8 was only measured in the static high magnetic field up to 25 T in Hefei for better signal. The s9 and s10 was only measured in PPMS for detecting Hall traces. It is noted that the results for the sample from different measurement systems are consistent and the log*B*-periodic oscillations appear with similar scale factor range in these different samples.

THEORETICAL DETAILS AND FURTHER DISCUSSIONS

It is suggested that log-periodic oscillations are closely related to the quasi-bound states of Weyl particles from the hole band with long-range Coulomb attraction when the carrier density is so dilute, and the long-range Coulomb attraction is generated by the charge impurity or the opposite type of carriers [1]. The Weyl equation with Coulomb attraction $V\left( \vec{R} \right)=\frac{-Ze^{2}}{4\pi\varepsilon_{0}R}$ obeys the scale invariance property [1, 2]. Here *Ze* is the central charge, and the fine structure constant $\alpha=\frac{e^{2}}{4\pi\varepsilon_{0}\hbar v_{F}}$ is larger than one due to the small Fermi velocity *v_F_* in Dirac materials. Thus, the so-called supercritical condition (*Z*$\cdot$*α* surpassing the angular momenta *κ*) can be matched in the system, and further gives rise to quasi-bound states solution in the system. In the following, we only consider the lowest angular momentum channel with* * =1. The combination of scale invariance and quasi-bound state solution results in the discrete scale invariance (DSI) property, and the radius of the quasi-bound states satisfy the relation $\frac{R_{n+1}}{R_{n}}=e^{\pi/{s_{0}}}$ with $s_{0}=\sqrt{\left( Z\cdot\alpha\right)^{2}-1}$ [1].

When a magnetic field is applied, the magnetic length $l_{B}=\sqrt{{\hbar c}/{eB}}$ is included, and the binding energy spectrum of the quasi-bound states evolve with the magnetic field. Using the Wentzel-Kramers-Brillouin method, our numerical simulation shows that the energy of the *n*-th quasi-bound states approaches the Fermi energy at the magnetic field *B_n_*, which obeys the approximate DSI property and the scaling ratio *λ* = *B_n+1_/B_n_* = $e^{{2\pi}/{s_{0}}}$ [1]. Aside from the quasi-bound states near the Coulomb center, large number of mobile carriers also exist in the lowest Landau level under the magnetic field in the ultra-quantum limit. Thus, the resonant scattering between the mobile carriers and the quasi-bound states around the Fermi level determine the transport properties of the material, e.g. the longitudinal MR and the Hall traces. Based on the t-matrix approximation [3], we derive the longitudinal conductivity beyond the quantum limit under large magnetic fields (the details are given in ref. 14 and the theoretical preprint [4]):

$\sigma_{xx}\left( \varepsilon_{F} \right)= \frac{4e^{2}}{h}l_{B}^{2}\left( n_{s}+n_{C}\frac{t^{2}}{8\pi\cdot\hbar v_{F}l_{*}^{-1}\cdot\Gamma\left( B \right)}\sum_{n} \frac{{\Gamma\left( B \right)}^{2}}{\left( \varepsilon_{F}-\varepsilon_{n}\left( B \right) \right)^{2}+{\Gamma\left( B \right)}^{2}} \right)$. (SE1)

Here *n_s_* is the density of short-range scatterers, *n_c_* is the density of Coulomb scatterers, *l_*_* is the effective length along the magnetic field, *t* is the coupling strength between the bound states with the continuum of the lowest Landau level, *ε_F_* is the Fermi energy, *ε_n_*(*B*) is the energy for the *n*-th bound state and (*B*) = *η^^* $\sqrt{B}$ is the width mainly determined by the broadening effect of the lowest Landau level with *η^^* depending on the microscopic scattering process and temperature [5]. The above microscopic formula can be further simplified into an empirical form which is more suitable for fitting the experimental data:

$\sigma_{xx}=\frac{4e^{2}}{h}l_{B}^{2}\left( n_{s}+n_{C}\frac{t^{2}}{8\pi\cdot\hbar v_{F}l_{*}^{-1}\cdot\Gamma\left( B \right)}\frac{\eta^{2}}{{sin}^{2}\left( \frac{s_{0}}{2}ln\left( \frac{B}{B_{0}} \right) \right)+\eta^{2}} \right)$, (SE2)

$\sigma_{xy}= \frac{4e^{2}}{h}l_{B}^{2}\left( N+n_{c}\frac{t^{2}}{8\pi\cdot\hbar v_{F}l_{*}^{-1}}\sum_{n} \frac{\varepsilon_{F}-\varepsilon_{n}\left( B \right)}{\left( \varepsilon_{F}-\varepsilon_{n}\left( B \right) \right)^{2}+{\Gamma\left( B \right)}^{2}} \right)$. (SE3)

Here, *N* denotes the total carrier density, *s*_0_, *B*_0_ and *η* are fitting parameters. In equation (SE2), the first term denotes the Anderson impurity scattering of the mobile carriers, which leads to linear-B dependent MR previously obtained by A. A. Abrikosov [6]; and the second term denotes the resonant scattering between the mobile carriers and the quasi-bound states, which gives rise to log-periodic correction to the MR. And the resistivity matrix elements can be obtained by the relation:

$\rho_{xx}=\frac{\sigma_{xx}}{\sigma_{xx}^{2}+\sigma_{xy}^{2}}$, (SE4)

$\rho_{yx}=\frac{\sigma_{xy}}{\sigma_{xx}^{2}+\sigma_{xy}^{2}}$. (SE5)

The correction to *ρ_xx_* is of the order *n_C_*/*n_S_*, while that to *ρ_yx_* is of the order *n_C_*/*N.* However, the full description of the background is hard to give due to complex band structure in HfTe_5_ because the carriers from trivial bands also contribute to this background but not to the oscillations (since the trivial bands are also beyond the quantum limit). Thus, although the DSI model cannot explain the saturation behavior of the magnetoresistance, the DSI model can give quantitative account for the log-periodic oscillations in our experiments, which is the main focus of our work.

The microscopic formula eq. (SE1) and the empirical formula eq. (SE2) can be used for fitting the experimental results. Figure S4 shows the fitting curves obtained with three fitting parameters *s*_0_, *B*_0_ and *η^^* for SE1* * for SE2. Here, *s*_0_ and *B*_0_ determine the energy for the *n*-th bound state *ε_n_*(*B*) in the microscopic formula eq. (SE1). At different temperatures, the thermal broadening effect changes the fitting parameters *η^^* or *η*. The fitting parameters are *s*_0_=4.8 (4.7) and *B*_0_=0.20 T (0.19 T) for sample s5 (s7). These parameters change slightly in different samples, which is consistent with the expectation that the DSI feature is mainly determined by fine structure constant in the material HfTe_5_.

The theoretical quantum magneto-oscillations in the Hall resistivity and the longitudinal resistivity can be obtained by formulas SE1, SE3, SE4 and SE5. The resulting curves are shown in Fig. S5, and the fitting parameters are *s*_0_=4.6 and *B*_0_=0.20 T. The peaks of *_xx_* correspond to the nodes of *_yx_*, and thus *_yx_* and *Δ**ρ_xx_* have a π/2 phase shift. The feature is similar to that in two-dimensional quantum Hall effect [7]. The π/2 phase shift can also be directly seen in the SE1 and SE3, in which the peaks of *σ_xx_* correspond to the nodes of *σ_xy_*. Thus, the resonant scattering between the mobile carriers and the quasi-bound states give rise to the log-periodic oscillations in both *_yx_* and *_xx_* with a π/2 phase shift. The experimental results of *R_xx_* and *R_yx_* in the same sample are shown in Fig.4. The experimental observations reveal that the phase of *R_yx_*is slightly ahead of *R_xx_*, and thus we conclude that the theoretical magneto-oscillations curves shown in Fig. S5 are consistent with the experimental observations.

In addition to the resonant scattering from the quasi-bound states around the Fermi energy, other scattering mechanisms also contribute to the magneto-transport with a non-oscillating background, which depends on the complex band structure of HfTe_5_ and cannot be explained in the DSI scenario. The theoretical formulas reveal that only the oscillations obey the DSI feature. Thus, the commonly used procedure of background subtraction for magneto-oscillations is utilized in our display [8].

The *R_xx_* curves shown in Fig. 2 and Fig. 3 in the main text increase, until the magnetic field reaches about 10 Tesla, and the *R_xx_* curves become slightly downward at around 20 Tesla. This trend is different from the case of ZrTe_5_. In ZrTe_5_ [1], the *R_xx_* increases with the magnetic field, and the slope becomes relatively flattened for magnetic fields larger than 5 Tesla, which may indicate that the trivial bands are localized with extremely large resistance, and only the Weyl band contributes to the resistance. In contrast, for the case of HfTe_5_, we speculate the trivial bands may behave differently from those trivial bands in ZrTe_5_, and one possibility is the formation of charge density wave for the trivial bands in HfTe_5_ leading to decreasing behavior of *R_xx_* [9]. The difference between ZrTe_5_ and HfTe_5_ may originate from different trivial band structures of these two materials, which needs further investigations. Moreover, it is noted that, despite the different trends of *R_xx_* in ZrTe_5_ and HfTe_5_, the log-*B* periodic oscillations from the Weyl bands are in common, owing to the universality of DSI in the Dirac materials.

REFERENCES

1. Wang H, Liu H and Li Y *et al*. Discovery of log-periodic oscillations in ultraquantum topological materials. *Sci Adv* **2018**; 4: eaau5096.
2. Ovdat O, Mao J and Jiang Y *et al*. Observing a scale anomaly and a universal quantum phase transition in graphene. *Nat Commun* **2017**; 8: 507.
3. Bastin A, Lewiner C and Betbeder O *et al*. Quantum oscillations of the Hall effect of a fermion gas with random impurity scattering. *J Phys Chem Solids* **1971**; 32: 1811-1824.
4. Liu H, Jiang H and Wang Z *et al*. Discrete scale invariance in topological semimetals. *arXiv*: 1807.02459.
5. Ando T and Uemura Y. Theory of quantum transport in a two-dimensional electron system under magnetic fields. *J Phys Soc Jpn.* **1974**; 36: 959-967.
6. Abrikosov AA. Quantum magnetoresistance. *Phys Rev B* **1998**; 58: 2788.
7. Wei HP, Tsui DC and Pruisken AMM. Localization and scaling in the quantum Hall regime. *Phys Rev B* **1986**; 33: 1488(R).
8. Schoenberg D. Magnetic Oscillations in Metals. Cambridge University Press, Cambridge, U.K., 1984.
9. Halperin BI. Possible states for a three-dimensional electron gas in a strong magnetic field. *Jpn J Appl Phys* **1987**; 26: 1913–1919.
